# Multiclass semantic segmentation for prime disease detection with severity level identification in Citrus plant leaves

**DOI:** 10.1038/s41598-025-04758-y

**Published:** 2025-07-01

**Authors:** P. Dinesh, Ramanathan Lakshmanan

**Affiliations:** School of Computer Science and Engineering, Vellore Institute of Technology, Vellore, India

**Keywords:** Deep learning, Computer vision, RSL Linked-TransNet, Multi-class segmentation, Prime disease classification and severity detection algorithm, Imaging and sensing, Computer science, Computational science, Engineering

## Abstract

Agriculture provides the basics for producing food, driving economic growth, and maintaining environmental sustainability. On the other hand, plant diseases have the potential to reduce crop productivity and raise expenses, posing a risk to food security and the incomes of farmers. Citrus plants, recognized for their nutritional benefits and economic significance, are especially vulnerable to diseases such as citrus greening, Black spot, and Citrus canker. Due to technological advancements, image processing and Deep learning algorithms can now detect and classify plant diseases early on, which assists in preserving crop health and productivity. The proposed work enables farmers to identify and visualize multiple diseases affecting citrus plants. This study proposes an efficient model to detect multiple citrus diseases (canker, black spot, and greening) that may co-occur on the same leaf. It is achieved using the RSL (Residual Squeeze & Excitation LeakyRelu) Linked-TransNet multiclass segmentation model. The proposed model stands out in its ability to address major limitations in existing models, including spatial inconsistency, loss of fine disease boundaries, and inadequate feature representation. The significance of this proposed RSL Linked-Transnet model lies in its integration of hierarchical feature extraction, global context modeling via transformers, and precise feature reconstruction, ensuring superior segmentation accuracy and robustness. The results of the proposed RSL Linked-TransNet architecture reveal average values of 0.9755 for accuracy, 0.0660 for loss, 0.9779 for precision, 0.9738 for recall, and 0.9308 for IoU. Additionally, the model achieves a mean F1 score of 0.7173 and a mean IoU of 0.7567 for each disease class in images from the test dataset. The segmentation results are further utilized to identify the prime disease affecting the leaves and evaluate disease severity using the prime disease classification and severity detection algorithm.

## Introduction

Agriculture is essential to the economy, especially in countries like India where it supports more than 70% of the farming population and accounts for roughly 17% of total GDP^1^. It is crucial for food security because it provides the nourishment needed to fight hunger, a problem that continues to plague underdeveloped countries^2^. In addition to providing jobs for more than 60% of the workforce, this agriculture supports the growth of a variety of crops that are essential for both domestic consumption and economic stability. Citrus plants are essential in the worldwide farming industry due to their considerable economic significance and impact on nutrition and health. They are extensively grown in over 52 countries, offering key nutrients such as vitamin C, which is crucial for human health^3^. The global production of citrus fruits, totalling around 157.98 million tons, highlights their importance in addressing food security with the increasing world population^4^. However, these citrus face severe damage by diseases like black spot, greening, and canker, which may result in serious monetary losses and decreased reliability of supplies^5^. Yields can be increased, and crop management improved by the early identification and categorization of these diseases utilizing cutting-edge technologies like deep learning^6^.

Effective detection of plant diseases is essential for successful agricultural practices, and it requires multiple stages of process. Initially, visual indicators such as variations in colour or markings on plant leaves are noticed; however, due to the complex symptoms and need for specific knowledge, this method can be tedious and inaccurate^7^. Deep learning models and other machine learning and computer vision techniques are used to increase accuracy in tasks like segmentation and classification on diseased images^1,8^. These existing models analyse leaf images to distinguish between areas that are healthy and areas that are unhealthy, making it possible to accurately diagnose various diseases. Preprocessing approaches for improved segmentation and feature selection lead to higher classification accuracies by improving the detection process^9^. Through these automated systems, quick identifications are conveyed, allowing farmers to implement essential actions to reduce crop loss. Effective preprocessing methods are essential for improving image analysis, especially in images of plant leaves. Major techniques involve Gaussian filtering and Region of Interest (ROI) extraction, utilized to reduce noise and concentrate on important aspects in the images^10^. Furthermore, histogram equalization is utilized to enhance colour distribution and improve feature retrieval, proving to be highly effective in low-quality images. Methods like Adaptive Histogram Equalization and Contrast-Limited Adaptive Histogram Equalization (CLAHE) enhance image quality even more in^2^.

Methods like semantic and instance segmentation are utilized to differentiate between both healthy and diseased regions on plant leaves, improving disease detection precision by examining texture, colour, and shape characteristics. Utilizing advanced deep learning models like Deep Overlay L-UNet enables precise identification of damage at the pixel level^4^. Furthermore, the effectiveness of the UNet-MBEO approach in segmentation tasks has been compared with other methods such as SegNet and K-means clustering. Multiclass segmentation involves identifying and outlining various categories within an image, commonly used in sectors like plant illness identification and medical visualizations^11^. demonstrated that the CAAR-UNet model effectively performed multiclass segmentation by identifying and segmenting different plant leaf diseases, resulting in high Intersection over Union (IoU) scores for specific classes such as northern corn blight disease and coffee leaves leaf miner.

This article presents a simple and efficient method for categorizing various classes in citrus plant leaves to detect the main disease and determine the severity level using deep learning techniques. The proposed approach will be validated on citrus plant leaf image data. Evaluation of the proposed method will be conducted using citrus plant leaves. The citrus leaf images are being transferred for preprocessing to enhance the data of the images. This high-quality dataset is used for pixel wise HSV (Hue Saturation Value) Multiclass masking and labelling to outline objects within an image. Once the data from the improved image dataset and the masked dataset with multiple classes are divided into training, testing, and validation sets, these will be used as input for the proposed model. The proposed RSL (Residual Squeeze & Excitation LeakyRelu) Linked-TransNet model is applied to segment different classes within an image. This seamless integration of hierarchical feature extraction, dynamic feature recalibration, and spatial reconstruction provides a superior model for multi-class semantic segmentation. By adjusting the thresholds for different classes within an image, this study can accurately determine the prime disease class and assess the severity of a diseased leaf based on pixel values across multiple classes with absolute precision. These disease types and their level of severity will be determined by examining the damaged leaf. In Sect. Related work, different methods for identifying plant diseases are discussed in the related works. Section Materials and methods outlines the proposed model, and Sect. Summary of experimental setup showcases the experimental findings. Meanwhile, Sect. Performance metrics gives performance metrics, Sect. Results and discussion provides the details related to the results and discussion, and Sect. Conclusion offers the conclusion along with references.

## Related work

Efficient plant disease detection and severity classification have become critical areas of research in agriculture due to their impact on crop yield and quality. Various studies have explored deep learning and computer vision approaches to address these challenges, integrating novel methodologies for improved performance^12^. propose SegLearner, a CNN-based segmentation framework for precise estimation of plant disease severity. Unlike traditional classification methods, SegLearner performs pixel-wise analysis to accurately distinguish healthy and diseased leaf areas. The model demonstrates superior performance in segmenting infection regions and quantifying severity. However, its accuracy may be affected by image quality, lighting variations, and overlapping disease symptoms.

For instance, Camellia oleifera, an economically significant crop, suffers from vulnerability to pests and diseases. To address this, the CTDUNet system was developed, incorporating a CNN base with Coordinate Space Attention and Cross-Attention mechanisms for enhanced alignment of image and text features. Hyperparameter optimization through grid search resulted in an average Intersection over Union (IoU) of 86.14%, outperforming other models^13^. Similarly, another approach segmented field images using the SAM framework, followed by leaf detection with Fully Convolutional Data Description (FCDD). This method achieved over 10% higher classification accuracy using PlantDoc and FieldPlant datasets^14^. The AgriDet framework advances plant disease severity classification through a Multi-variant Grabcut Algorithm for affected region detection, Gaussian Mixture Models for feature separation, and a Kohonen Learning Layer for multi-scale feature extraction. Achieving a validation accuracy of 96%, this model classifies disease severity into four levels^15^. Another innovation, PlantDet, combines models like InceptionResNetV2 and EfficientNetV2L to tackle underfitting and overfitting issues. Tested on the Rice Leaf dataset, it surpassed benchmarks with a precision of 98.50% and a specificity of 99.71%^16^.

Building on this, AgriSegNet leveraged multiscale attention-based semantic segmentation for UAV-acquired farmland images, enhancing anomaly detection in agricultural planning^17^. Moreover, hyperspectral image segmentation methods, such as MCCA and PCCA, have improved feature extraction and dimensionality reduction, demonstrating superior accuracy compared to traditional PCA techniques^18^.

In crop-specific studies, CONF-RCNN—a two-stage model combining Faster R-CNN and conformer-based layers—proved effective for tomato leaf disease detection, achieving 90% accuracy on real-world datasets^19^. Another approach employed an enhanced Pyramid Scene Parsing Network (PSPNet) with auxiliary classification loss for pixel-level segmentation, achieving improved Dice Similarity Coefficient (DSC) scores by integrating diverse datasets^20^.

Several models have further refined segmentation and classification techniques. The DIResUNet framework incorporates a dense global spatial pyramid pooling (DGSPP) module for better feature extraction, excelling in high-resolution remote sensing applications^21^. Similarly, ESDINet introduced a lightweight dual-branch network for multiscale interaction, achieving high mIoU and processing speeds^22^. IoT integration has also been explored for real-time disease diagnosis. A system combining ICS, MSO, and MO-DNN algorithms achieved 97.3% accuracy, facilitating quick results transmission to farmers via mobile devices^23^. Techniques like MC-SVM have been employed for feature extraction and disease classification, with frameworks achieving up to 95.44% accuracy on PlantVillage datasets^24^.

Advanced image manipulation methods, such as the TRL-GAN model, further enhance classification accuracy by generating realistic diseased leaf images. With ResNeXt101 achieving 97.45% accuracy, the integration of TRL-GAN yielded a notable improvement in performance^25^. Finally, lesion segmentation using weighted techniques and comprehensive feature extraction has achieved high accuracy rates across various datasets, particularly for citrus plant disease detections.

### Semantic segmentation on plant leaves

Semantic segmentation, a critical technique in computer vision, labeling involves each and every pixel in an image with a specific category, enabling a detailed understanding of image contents (J Jiang et al., 2023)^7^, . This field encompasses single-class and multi-class segmentation, with the latter aiming to distinguish multiple classes within an image. Modern semantic segmentation architectures, such as Fully Convolutional Networks (FCN), U-Net, SegNet, and DeepLab, are designed to handle images of varying sizes while preserving spatial dimensions for accurate segmentation. Among these, U-Net, with its encoder-decoder layout and skip connections, excels at capturing both local and global context.

Recent advancements in segmentation models highlight their diverse applications and enhancements. For instance, DeepLabv3 + employs data augmentation to improve rust transmission hub identification^27^. sAddressing specific challenges in cotton leaf analysis, GlandSegNet leverages an encoder-decoder architecture and an ECA attention module to detect pigment glands with superior accuracy, achieving area accuracy rates of 0.9842 and 0.9510 on two datasets^28^.

MCC-Net introduces innovative components such as multi-scale convolutional blocks and attention modules, achieving a mean IoU of 92.56% and a dice coefficient of 96.33%, outperforming existing models^29^. Similarly, MTLSegFormer integrates multi-task learning with attention mechanisms, dynamically weighting image regions based on task relevance, while MTS-CNN incorporates crop, weed, and combined loss calculations, demonstrating superior performance on multiple datasets^30,31^. To enhance dataset quality, a method utilizing conditional GANs (cGAN) generates semi-artificial samples by replacing objects in real images, creating realistic four-channel multi-spectral images^32^.

^33^ present an improved ResNeXt model for diagnosing fungal diseases in apple crops. By enhancing the architecture to better capture leaf-specific features, the model achieves over 95% classification accuracy on a preprocessed, heterogeneous dataset. Key preprocessing steps include image enhancement to normalize lighting and reduce noise. While results are promising, performance may decline under extreme conditions like heavy shadowing or overlapping leaves, highlighting the need for further real-world validation. The modified ResNeXt model was trained on this preprocessed^6^. The experimental results demonstrated that the proposed model achieved a classification accuracy exceeding 95% across multiple crop disease categories. The model’s performance can degrade under extremely noisy or occluded conditions, such as overlapping leaves or extreme shadowing, which the dataset might not fully represent.

Deep learning models like U-Net, SegNet, and DDCN have proven effective for segmenting citrus trees in multispectral images, with DDCN excelling in shadow-affected areas^34^. UAV imagery combined with a U-Net model showed promising results for tobacco plant segmentation, with accuracy varying by flight height and optimal performance observed at 50 m^35^. Furthermore, the introduction of the WE3DS dataset for RGB-D image segmentation in crop farming has shown that incorporating depth information significantly enhances segmentation accuracy, achieving a mean IoU of 70.7%^36^. Transformer-based models are also gaining traction, as demonstrated by Swin-Unet, which incorporates multi-channel information and self-attention mechanisms. This model improved segmentation performance across 80% of regions and increased the average IoU by 0.19^37^. These advancements showcase the continuous evolution of semantic segmentation techniques, driven by novel architectures and tailored datasets.

Existing models in plant disease detection and severity classification, while innovative, often face limitations in key areas. Many approaches shows difficulty in handling diverse datasets effectively, underperforming in complex real-world scenarios involving varying illumination, occlusions, and overlapping regions. Persistent challenges such as overfitting due to inadequate data augmentation, lack of multi-modal feature integration, and computational inefficiency in large-scale applications further hinder their performance. Although deep learning architectures like U-Net and SegNet show promise, they often fall short in precisely addressing pixel-level segmentation across varying crop types. Additionally, semantic segmentation techniques frequently neglect certain important contextual information and fail to achieve optimal feature alignment in multi-class settings.

By overcoming these challenges, the RSL Linked-TransNet shows its significance in handling diverse image regions, emphasizing relevant features, and maintaining fine details across complex datasets. Its seamless integration of hierarchical feature extraction, dynamic feature recalibration, and spatial reconstruction establishes it as a superior model for multi-class semantic segmentation.

**Fig. 1:**
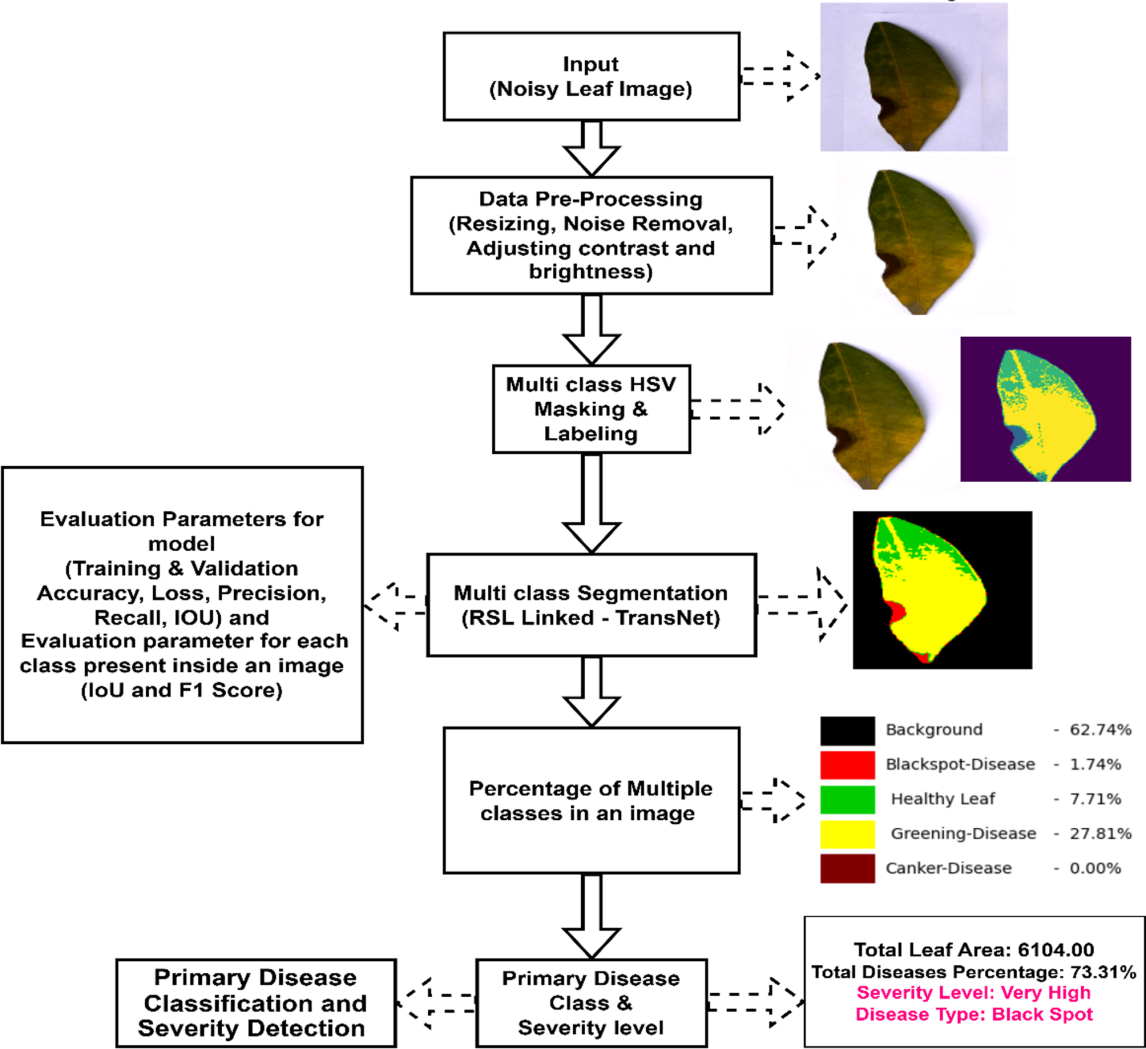
The Proposed Systems Architecture.

## Materials and methods

This section gives a summary of the dataset and methods employed for pixel wise segmenting various diseases in an images of citrus plants. Firstly, the dataset considered for this work is discussed in this section, followed by a brief overview of the image pre-processing step. An effective method for annotating pixel wise Multi-class HSV masking and labelling method for in every image data from the pre-processed dataset. Subsequently, the proposed method of pixel wise multi-class semantic segmentation with RSL Linked-TransNet and its nature will be discussed. This method aims to predict the percentage of severity for multiple classes within an image, as well as predict the main disease class and its severity level. Figure 1 shows the proposed system architecture along with the flow.

### Citrus disease leaf dataset

**Fig. 2:**
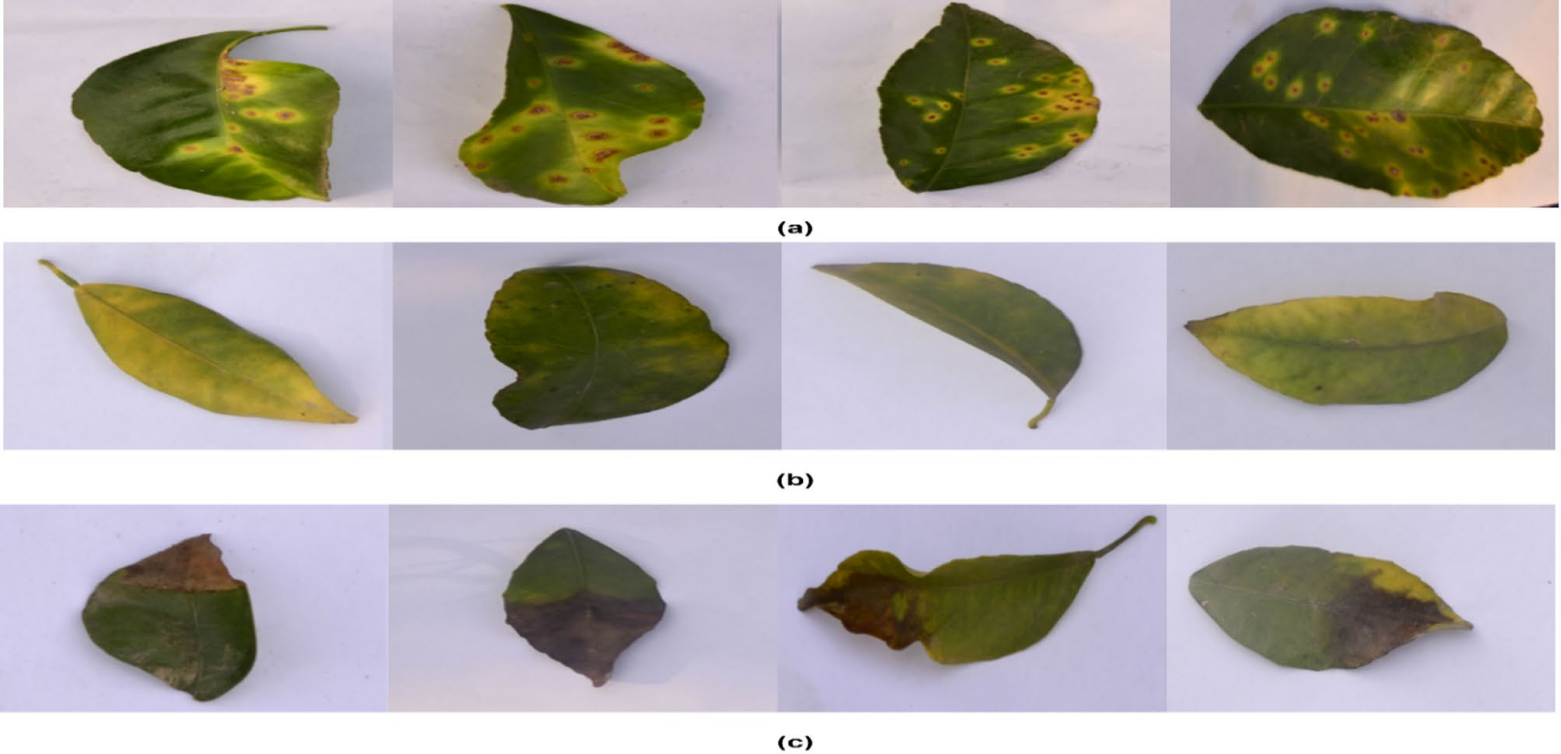
Samples from Citrus Disease Dataset: (**a**) Noisy citrus canker images, (**b**) Noisy citrus greening disease images, (**c**) Noisy citrus blackspot images.

The proposed work make use of the publicly accessible citrus leaves setup dataset^38^. The dataset includes pictures that have a spatial resolution of 256 × 256. Three of the most frequently observed citrus diseases are black spot, greening, and canker. The dataset is smaller in size and diminished in quality. The sample images from the dataset are shown in Fig. 2.

### Pre-processing

**Fig. 3:**
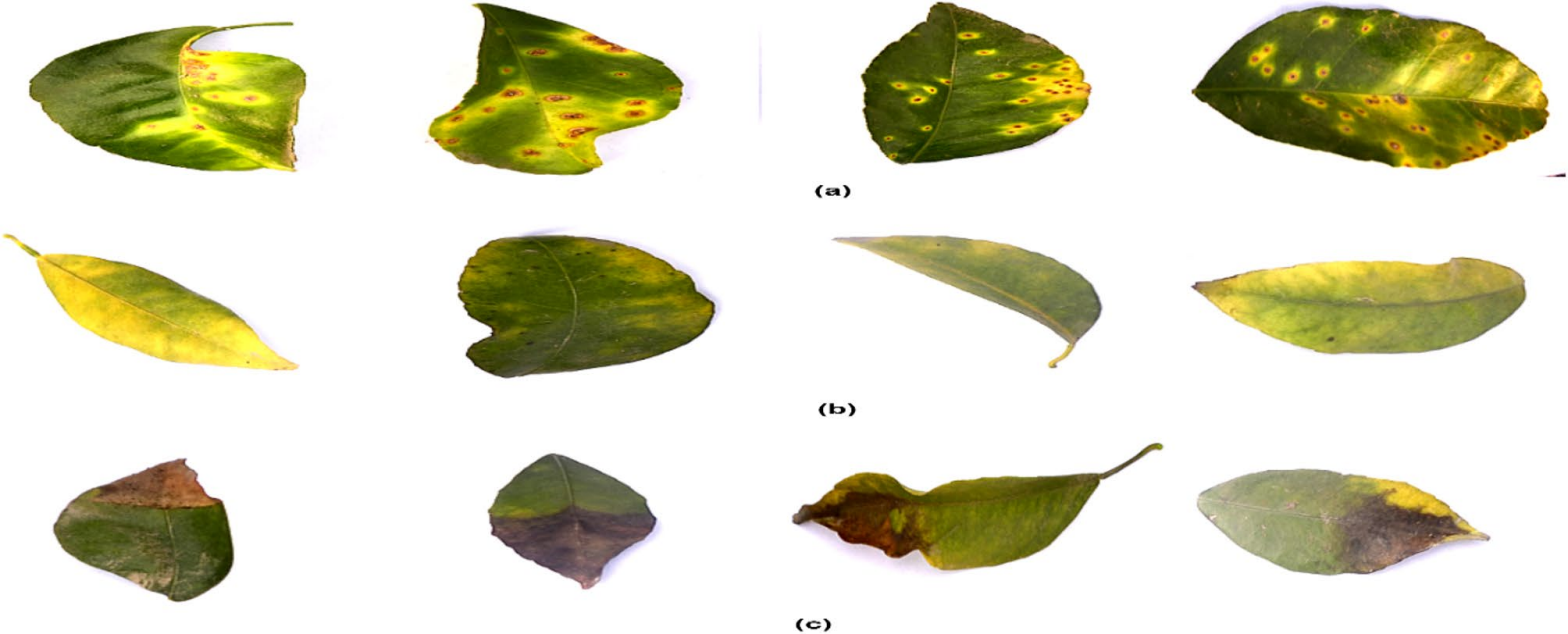
Sample Pre-Processed Images Citrus Disease: (**a**) Enhanced citrus canker images, (**b**) Enhanced citrus greening, (**c**) Enhanced citrus blackspot images.

Further this research aims to improve the accuracy of disease detection in plant pathology by enhancing preprocessing techniques for citrus leaf image quality. The first step of preprocessing is crucial because it changes basic images into a format that is more convenient for analysis^39^. This process includes several enhancements: increasing contrast by 10% to emphasize features, enhancing brightness by 60% to address potential underexposure, and tripling sharpness to highlight finer details^40^. These adjustments are crucial to ensure that future machine learning models can accurately identify and utilize the pixel-level fundamental elements of the data. The improved sample images from the dataset are shown in Fig. 3.

### Multi-class HSV masking and labelling for pixel specific mask

This procedure enables the division and categorization of various pixel areas in images of citrus leaves, improving the multiclass examination of diseases such as citrus canker, blackspot and greening in an image.

In addition, to establish the HSV colour range for mask each pixel in leaf disease image, begin by taking high-quality pre-processed images of the diseased leaves. Use OpenCV to change these pictures from RGB to HSV colour space. Determine the specific pixel areas in the HSV images that are of interest region and select the HSV values of the affected pixel areas that are close to the colours yellow, green, and brown in the HSV colour space. Establish the minimum and maximum thresholds limits for these HSV values in order to generate pixel wise masks mapping. The designated colour ranges for citrus canker and blackspot: the lower brown and the upper brown, for citrus greening: the lower yellow and the upper yellow, and for a healthy citrus leaf: the lower green and the upper green, these limits are shown in Table 1. Masks of different diseases are made by different colours using CV2.inRange and then merged to create a single mask that mask pixel specific areas.

**Table 1:** Threshold colour ranges for diseases.

Disease Classes	Threshold colour Limits		Hue	Saturation	value
Canker, Blackspot	Brown Limits	Lower limit	1	0	0
		Upper limit	19	255	255
Greening	Yellow Limits	Lower limit	20	25	0
		Upper limit	29	255	255
Healthy	Green limits	Lower limit	30	18	0
		Upper limit	100	255	255

**Fig. 4:**
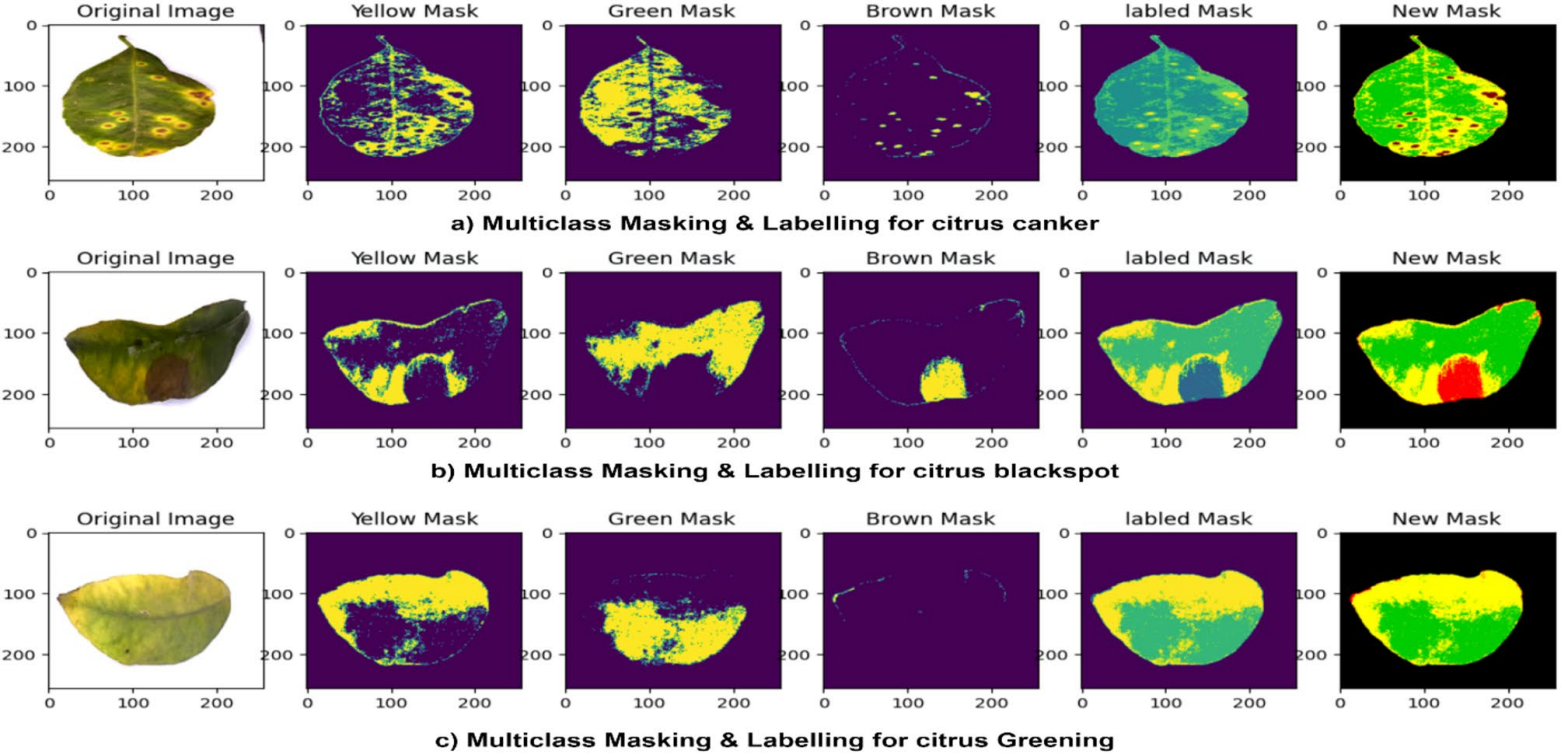
Sample images of the process of multiclass masking and labelling in citrus leaves diseases.

To label the region wise pixels in an image, a mask is generated for the pixel areas not covered by the main mask, and different labels are applied to each pixel area threshold limits, specific disease are given to five different labels: 4 represents canker, 3 represents greening, 2 represents healthy, 1 represents blackspot, and 0 represents background. Region wise pixels in an image is marked and labelled masks are then created and subsequently stored as mask image, along with the original image. The threshold limits and labels are shown in Table 2. These process of the multi-class HSV masking and labelling for creating mask dataset are shown in the Fig. 4.

**Fig. 5:**
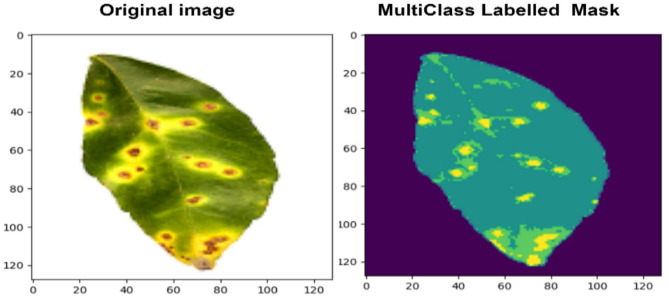
Sample Input and Masked Image from Dataset for Multi-Class Segmentation.

**Table 2:** Labelling used for different classes.

Disease Classes	Threshold colour limits	Labels
Canker	Brown	4
Blackspot	Brown	1
Greening	Yellow	3
Healthy	Green	2
Background	Non-Masked region	0

Datasets are specifically created for pixel wise multiclass disease detection by utilizing the initial image and labelled image, which enables the segmentation and categorization of various classes in citrus leaf images and improves the assessment of diseases. Sample input image and its multiclass masked image from the dataset for multi-class semantic segmentation is shown in Fig. 5. The created multiclass disease segmentation dataset contains 516 image and 516 mask and resized into 256 $\:\times\:$256. In this each images mask that contains pixel wise region map of five classes and as mentioned in Table 3, dataset is spitted for train, test, validation set.

**Table 3:** Dataset split, and number of images used in train, test and validation.

No. of images					
Train set		Test set		Validation set	
Image	Mask	Image	Mask	Image	mask
412	412	52	52	52	52

### Proposed methodology

**Fig. 6:**
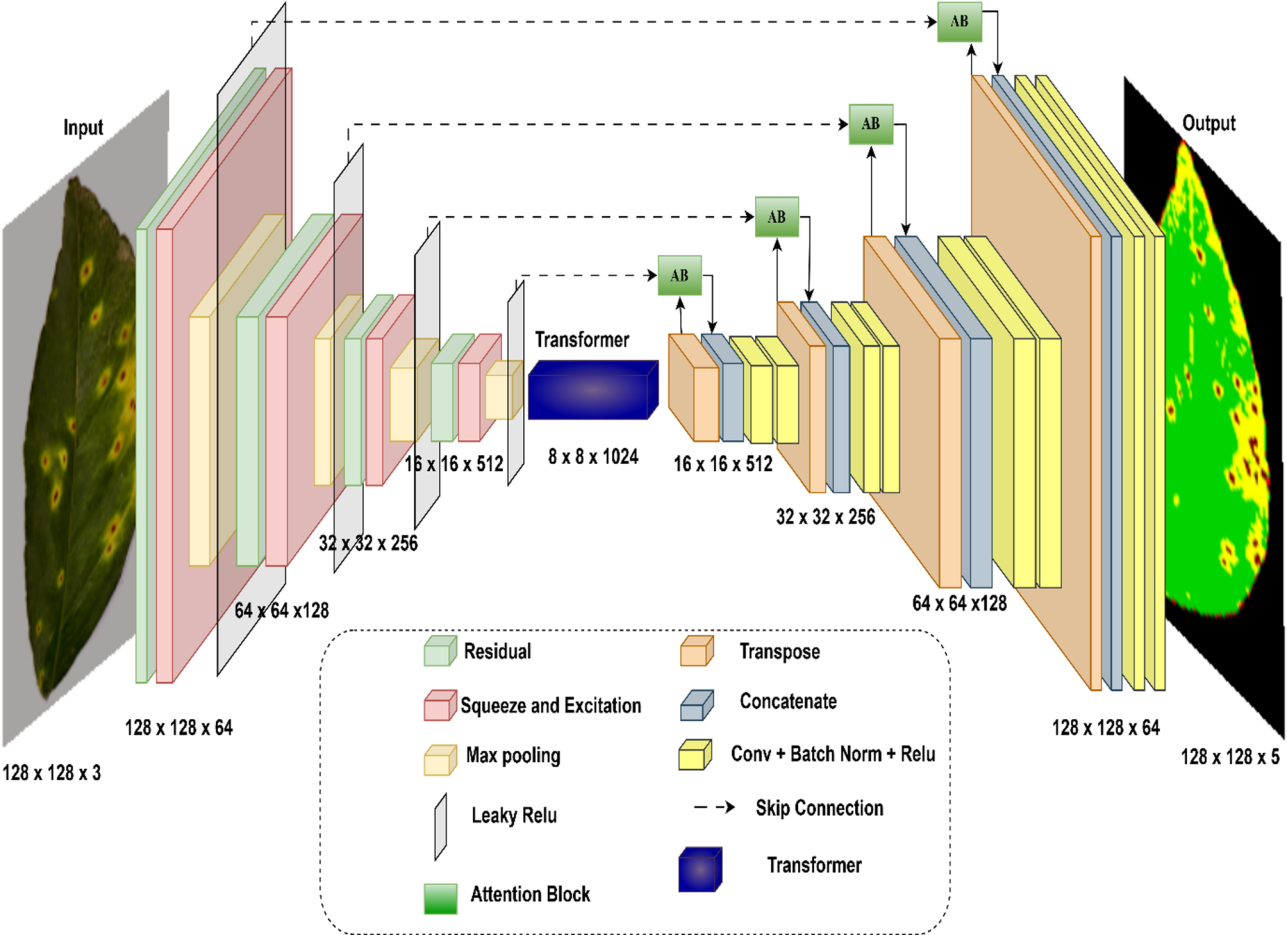
Architecture diagram of RSL Linked-TransNet model.

The architecture of the RSL Linked-TransNet is carefully designed to ensure a seamless flow of information across all stages of the model, from input to output, enabling precise and efficient multi-class semantic segmentation. Each component of the architecture contributes to the progressive extraction, transformation, and reconstruction of disease leaf image features while maintaining spatial and contextual coherence. Figure 6 displays the proposed architecture diagram for RSL Linked-TransNet.

The model begins with an input image $\:X\: \in \:{\mathbb{R}}^{H\times\:W\times\:C}$, where $\:H$ is the Image height. $\:W\:$is the Image width and $\:C$ is the Number of input channels 3 for RGB images. The model goal is to produce a pixel-wise segmentation map $\:\widehat{Y\:}\: \in \:{\mathbb{R}}^{H\times\:W\times\:{F}_{O}}$, where $\:{F}_{O}$​is the number of output classes. The transformation is represented as: $\:\widehat{Y\:}=Softmax\:\left({w}_{out}^{1\times\:1}*{T}_{4}\right)$.

Where $\:{T}_{4}$ is the refined feature map from the final decoder block and$\:{w}_{out}^{1\times\:1}$ represent the learnable weights of a $\:1\times\:1$ convolution. Figure 6 displays the proposed architecture diagram for RSL Linked-TransNet.

The encoder path extracts hierarchical features from the input disease leaf image, progressively reducing the spatial dimensions while increasing the feature richness. Each encoder block generates two outputs: the skip connection features $\:{Skip}_{i}$ and the downsampled features ​$\:{Pool}_{i}$. The encoder block is defined as:

1 $$\:{Pool}_{i},\:{Skip}_{i}={Encoder}_{i}\:\left(X\right)=\left(SE\:\left(Residual\:\left(X,\:{F}_{i}\right)\right),\:MaxPool\:\left(SE\:\left(Residual\left(X,{F}_{i}\right)\right)\right)\right).$$

Each block plays a specific role: The encoder path begins with residual blocks and (Squeeze-and-Excitation) SE blocks, ensuring that critical image features are captured and enhanced through channel-wise recalibration.

In multi-class segmentation, fine details like boundaries of small disease classes can be lost in deeper layers. Residual connections help retain these disease details by allowing gradients to flow directly through the network. The residual block alleviates the vanishing gradient problem during training by introducing skip connections that preserve both low- and high-level spatial details.

2 $$\:Residual\:\left(X,F\right)=ReLU\:\left(BN\left({W}^{3\times\:3}*X\right)+\:{W}_{shortcut}^{1\times\:1}*X\right).$$

Here, $\:{W}^{3\times\:3}$: are the weights of a $\:3\times\:3$ convolution, $\:{W}_{shortcut}^{1\times\:1}$ is weights of a $\:1\times\:1$ convolution for dimensions alignment and $\:BN$ is batch normailzation. Process in multi-class segmentation, certain feature channels might be hard for distinguishing between classes like differentiating diseases and background. SE blocks improve the representation by highlighting these features. The SE block captures the importance of channel-wise features by modeling their interdependencies on multiple classes. It selectively emphasizes informative channels while suppressing irrelevant ones. In multi-class segmentation, varied intensity values in images like shadowed or dark regions in plant leaf can lead to small or negative activations.

3 $$\:SE\left(X\right)=X\bullet\:\:\sigma\:\:\left({W}_{dense2}\:\bullet\:ReLU\:\left({W}_{dense1}\:.\:GAP\left(X\right)\right)\right).$$

Where $\:GAP\left(X\right)$ denotes Global average pooling of X, $\:{W}_{dense1}\:,\:\:{W}_{dense2}$​ are Dense layer weights and $\:\sigma\:$: Sigmoid activation function. Additionally, Leaky ReLU ensures that these features are not entirely ignored. Leaky ReLU allows a small gradient flow for negative inputs, preventing neurons from dying on zero gradient issue. Max pooling helps capture the global structure of the leaf image, reduces the spatial dimensions of the feature map, focusing on the most prominent features within a leaf region.

The transformer block at the bottleneck global feature extraction and long-range dependencies are captured using transformers. By leveraging multi-head attention, the model captures long-range spatial relationships, essential for segmenting objects of the same disease class scattered across the image. It ensures that these regions are connected.

4 $$\:{T}_{b}=Transformer\:\left({Pool}_{4}\right)=\:{W}^{3\times\:3}*ReLU\:\left(LayerNorm\left(X\right)+MultiHeadAttention\left(X,X\right)\right).$$

Here, $\:LayerNorm$ normalize inputs, $\:MultiHeadAttention$ captures the long-range dependencies and $\:{W}^{3\times\:3}$​ are convolutional weights refining the transformer output. Layer normalization and residual connections stabilize the flow of features through the transformer, ensuring that global dependencies are effectively captured without disrupting the learned representations.

The decoder path progressively reconstructs the segmentation map, restoring spatial dimensions and utilizing encoder features via skip connections, progressively upsampling features using transposed convolutions. It restores spatial dimensions for pixel-level segmentation.

5 $$\:{T}_{j}={Decoder}_{j}\left(\:{T}_{j-1},\:{Skip}_{4-j}\right)={W}_{conv}^{3\times\:3}*Concat\:\left[{\:T}_{j-1},\:Attention\:\left({\:T}_{j-1},\:{Skip}_{4-j}\right)\right].$$

Attention blocks further refine the skip connections by weighting and aligning relevant features from the encoder with the upsampled decoder features.

6 $$\:Attention\:\left(U,Skip\right)=U.\:\sigma\:\:\left(\:{W}_{att}^{3\times\:3}*ReLU\:\left(\:{W}_{att}^{3\times\:3}*Skip+\:{W}_{att}^{3\times\:3}*U\right)\right).\:$$

$\:U$ represents Upsampled features, $\:Skip$ are skip connection features and $\:{W}_{att}^{3\times\:3}\:$are Learnable attention weights. Decoder ensures that fine disease details, such as disease boundaries, are accurately reconstructed. By concatenating skip features and refining them with convolutional layers, the decoder has high-resolution spatial details are preserved in the reconstruction process.

The final step applies a $\:1\times\:1$ convolution to reduce the channel dimensions to $\:{F}_{O}$​ number of output classes followed by softmax activation function.

7 $$\:\widehat{Y\:}=Softmax\:\left({w}_{out}^{1\times\:1}*{T}_{4}\right).$$

**Fig. 7:**
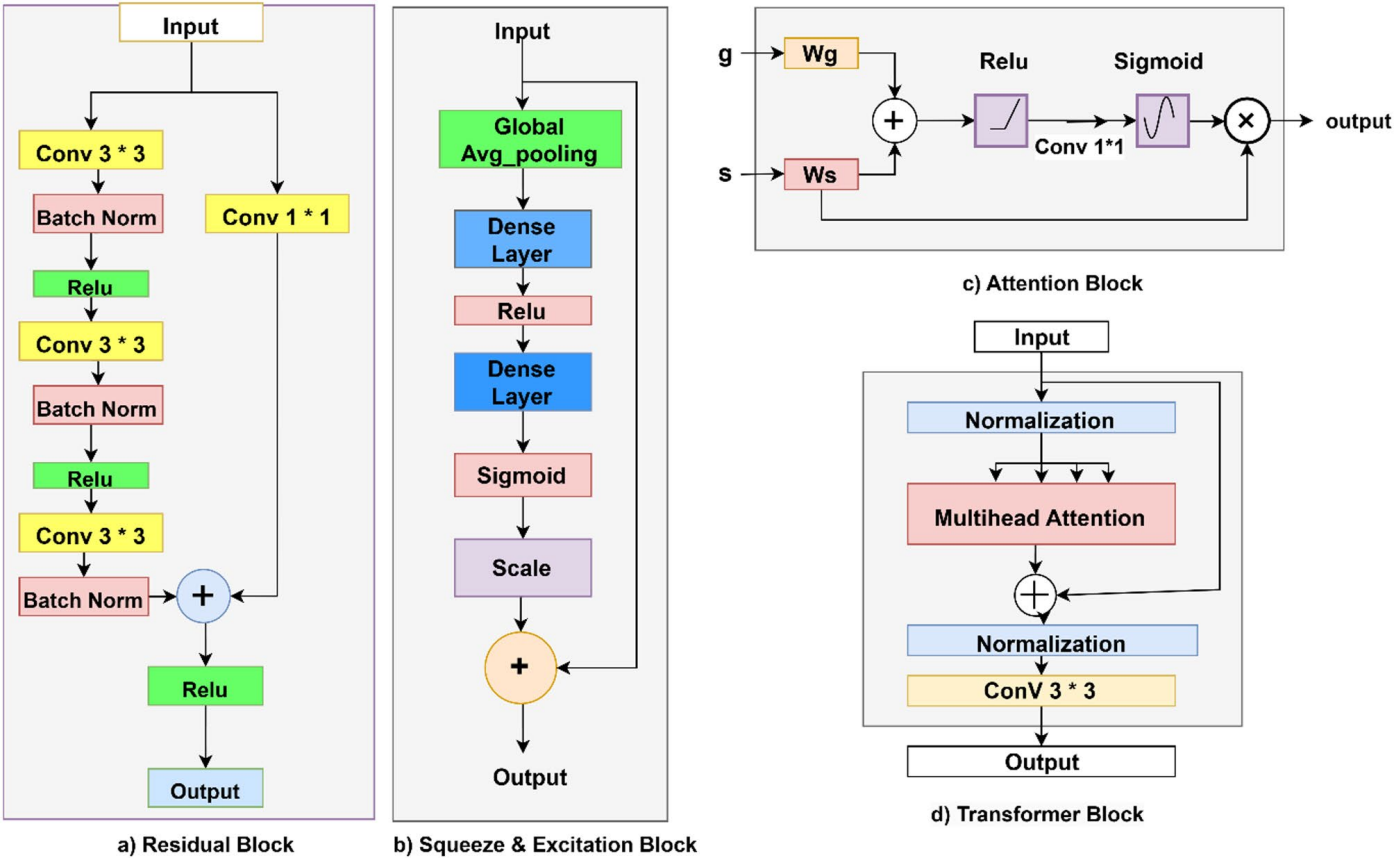
Details of the essential blocks that are in Multiple class Segmentation model.

The softmax activation layer ensures a seamless mapping of the refined feature maps to pixel-wise class probabilities assigning to one of the target disease classes, producing a precise and coherent segmentation map. This integration ensures efficient feature representation and accurate segmentation for complex disease detection tasks. Figure 7 illustrate the specifics of the key blocks that are present in it.

The combination of hierarchical feature extraction from encoder, global context modeling from bottleneck, and precise feature reconstruction in decoder represents the novelty of this paper, that ensures the RSL Linked-TransNet: Preserves spatial consistency: Through skip connections, attention mechanisms, and residual pathways. Handles diverse image regions: By emphasizing relevant features via SE blocks and attention blocks. Produces accurate segmentation maps: By integrating refined global and local features at each stage. This seamless flow from input to output ensures that the model effectively segments multi-disease classes in images with high precision, achieving robust performance across a variety of challenging data.

### Prime disease classification and severity detection

This research focuses on assessing the severity extent of each disease class on a leaf by calculating the proportion of the disease-area and identifying those prime disease causing the damage by using the proposed models result. The combined disease percentage is calculated to represent the cumulative area affected by specific disease classes. Let $\:{C}_{disease}$ ​ be the set of disease related classes {1, 3, 4} are Blackspot, Greening and Canker. $\:{C}_{leaf}$ is a Set of all relevant classes, including healthy {2} and diseased areas {1, 3, 4}. The combined disease percentage is given by:

8 $$\:{P}_{disease}=\:\frac{\sum\:{i\in\:C}_{disease}\:{N}_{{class}_{i}}}{\sum\:{i\in\:C}_{leaf}{N}_{{class}_{i}}}\times\:100.$$

Here, $\:{P}_{disease}$ indicates the overall proportion of the leaf affected by diseases, relative to the sum of pixels belonging to all relevant leaf classes $\:{C}_{leaf}$.

The severity of a leaf disease is evaluated by determining the percentage of the affected area. $\:{P}_{disease}$ and comparing it to predefined thresholds. Based on this comparison, the severity levels are classified as follows:

1. Very Severe Disease-Affected Leaf: If $\:{P}_{disease}$ exceeds the threshold for severe disease $\:{T}_{highly\:severe}$ is 40% and above, the leaf is considered very severely affected. This indicates extensive damage and requires immediate attention.
2. severe Disease-Affected Leaf: If $\:{P}_{disease}$​ lies between the threshold for severe disease $\:{T}_{severe}$ is 30% and $\:{T}_{highly\:severe}$​, the leaf is classified as severely affected. This level suggests significant but not extreme disease progression.
3. Average Disease-Affected Leaf: If $\:{P}_{disease}$​ is less than or equal to $\:{T}_{severe}$​, the leaf is categorized as having an average level of disease. This indicates mild damage that may not immediately require intervention.

#### Disease classification

The class-wise percentage distribution is calculated by determining the percentage of pixels in an image that belong to each class like diseases or background. Given the total number of pixels in the predicted mask $\:{N}_{total}$​, the count of pixels for each class $\:{N}_{{class}_{i}}$ is obtained using:

9 $$\:{P}_{{class}_{i}}=\:\frac{{N}_{{class}_{i}}}{{N}_{total}}\:\times\:100.$$

Here, $\:{P}_{{class}_{i}}$ ​​ represents the percentage of pixels for the $\:i$-th class, providing insight into the distribution of each class in the segmented image. Disease classification involves identifying the type of disease affecting the leaf based on predefined thresholds for disease-related classes. The classification process follows these rules:

1. If the percentage of pixels for the Canker class $\:\:{P}_{Canker}$exceeds a predefined threshold $\:\:{T}_{Canker}$, the leaf is classified as affected by Canker.
2. If $\:\:{P}_{Canker}$is below the threshold, but the Blackspot class percentage $\:\:{P}_{blackspot}$exceeds its threshold $\:\:{T}_{blackspot}$, the leaf is classified as affected by Blackspot.
3. If both $\:\:{P}_{Canker}$and $\:\:{P}_{blackspot}$are below their respective thresholds, but the Greening class percentage $\:\:{P}_{greening}$exceeds its threshold $\:\:{T}_{greening}$, the leaf is classified as affected by Greening.
4. Otherwise, the leaf is classified as healthy

**Fig. 8:**
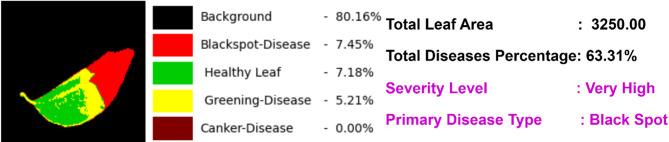
Visualize the segmentation results, each class percentage, severity level and prime disease type.

The thresholds $\:\:{T}_{Canker}$is 0.1​, $\:\:{T}_{blackspot}$is 1, and $\:\:{T}_{greening}$ is 9 are disease-specific to our dataset. Figure 8 presents the segmentation results, from which the percentage of each class, the severity type, and the classification of the prime disease are derived.
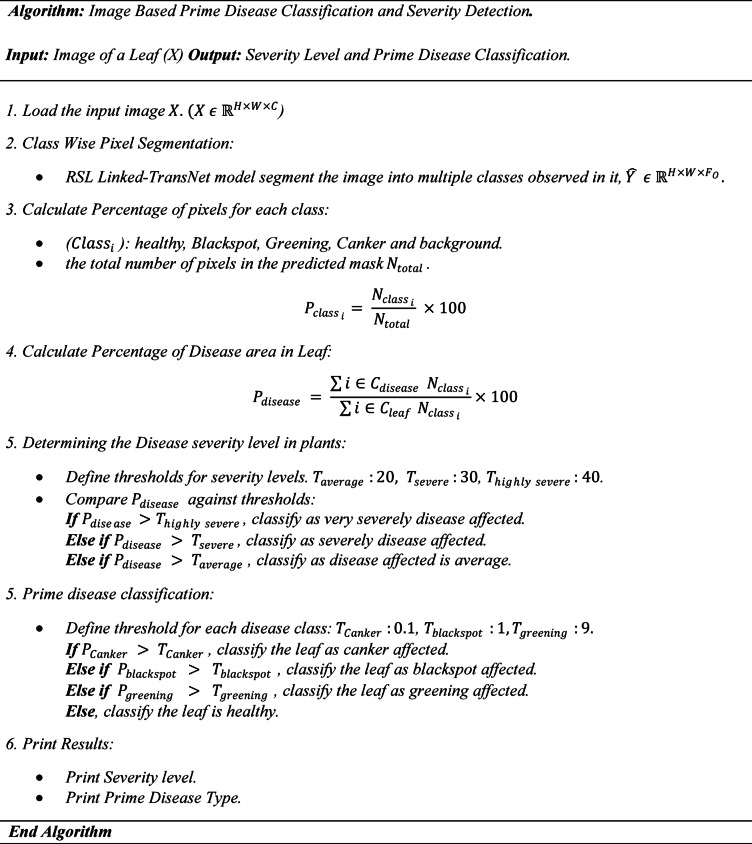


## Summary of experimental setup

This study presents an end-to-end deep learning approach for multi-class semantic segmentation and severity classification of citrus leaf diseases.

**Fig. 9:**
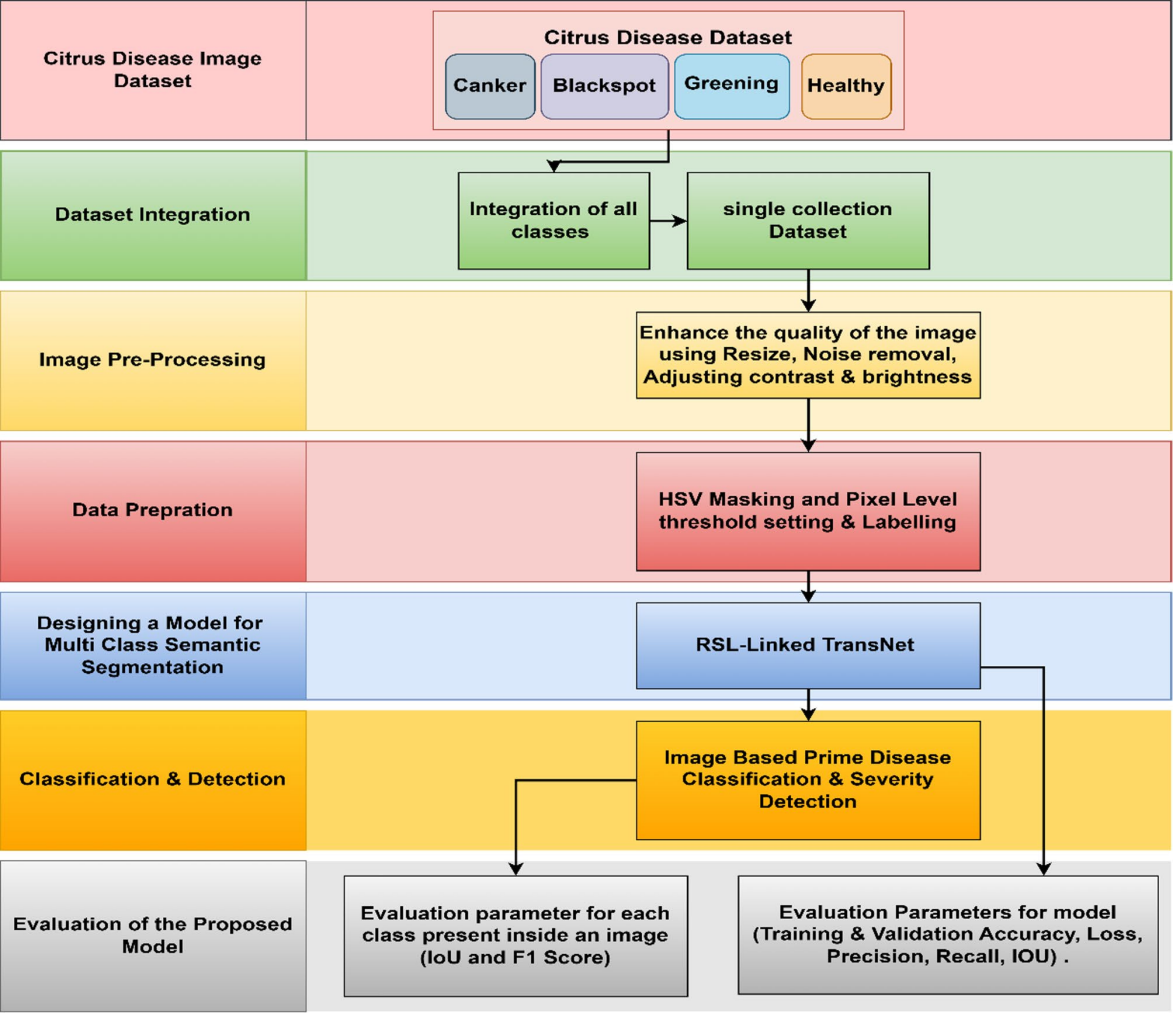
A High-Level Workflow Diagram.

As illustrated in Fig. 9, the dataset consists of annotated images across four categories: Canker, Blackspot, Greening, and Healthy leaves. Preprocessing steps included image resizing, noise removal, and contrast enhancement. HSV masking and thresholding were used to generate precise pixel-level ground truth masks. The proposed segmentation model, named RSL-Linked TransNet, accepts 128 × 128 × 3 inputs and predicts five classes, including the background. It was trained using the Adam optimizer with a learning rate of 0.0001, categorical cross entropy loss, and Early Stopping was used. Performance was observed using metrics like Accuracy, IoU, Dice score, AUC, Precision, and Recall.

After training, predictions were made on test images using the trained model. Each prediction was visualized using class-wise RGB colour codes, displaying the real input image, the ground truth segmentation, the predicted mask, and a coloured version for insightful understanding. Pixel-wise performance metrics such as per-class IoU were computed to assess segmentation quality. In addition to segmentation accuracy, the model outputs were used to calculate the area percentage affected by each disease class. Based on those values, a severity level classification was implemented using the Image Based Prime Disease Classification and Severity Detection algorithm, further by applying defined thresholds, each leaf was categorized into severity levels such as Average, Heavily, or Very Severely Affected. Furthermore, the dominant disease (Canker, Blackspot, or Greening) was inferred from the percentage presence of the respective class in the prediction. Healthy leaves were also accurately identified when disease class percentages were negligible. Performance of the prime disease classification is measured by IoU and F1 score. This end-to-end pipeline not only ensures precise semantic segmentation but also provides an interpretable framework for real-time disease monitoring and severity assessment in agricultural contexts.

## Performance metrics

This section outlines the key performance metrics used to evaluate the segmentation models, focusing on accuracy, loss, and several class-level and pixel-level indicators. Each metric provides a unique perspective on the model’s prediction quality and its ability to distinguish and classify leaf disease features accurately.

### Accuracy

Accuracy measures the proportion of correctly predicted labels to the total predictions.

10 $$\:Accuracy\:=\:\frac{No.\:\:of\:Correct\:Predictions}{Total\:Number\:of\:Predictions}.$$

Number of Correct Predictions: The count of predictions made by the model that match the actual ground truth. Total Number of Predictions is the total count of predictions made by the model.

### Loss

The categorical cross-entropy loss function, a widely used metric for multi-class classification tasks, quantifies the difference between the true labels and the predicted probabilities, penalizing incorrect predictions more as they deviate further from the ground truth. Where $\:N$ is the total number of pixels, $\:C$ is total number of classes, $\:{Y}_{i,c}$ is ground truth label for class $\:c$ at pixel $\:i$ and $\:{\widehat{Y\:}}_{i,c}$ is predicted probability for class $\:c$ at pixel $\:i$.

11 $$\:Loss=\:-\:\frac{1}{N}\sum\:_{i=1}^{N}\sum\:_{c=1}^{C}{Y}_{i,c}\text{log}\left(\:{\widehat{Y\:}}_{i,c}\right).$$

### Intersection over union (IOU)

Assesses the intersection of the anticipated segmentation and the actual results.

12 $$\:IoU\:=\:\frac{Intersection}{Union\:+\:\epsilon}\:=\:\:\frac{\sum\:(Y\:.\:\:\widehat{Y\:})}{\sum\:Y+\:\sum\:\widehat{Y\:}-\sum\:(Y\:.\:\:\widehat{Y\:})}.$$

It measures overlap between the predicted segmentation and the actual ground truth.

### Dice coefficient

Measures the similarity between the predicted segmentation and the ground truth.

13 $$\:Dice\:Coefficient\:=\:\frac{2\:\times\:\:Intersection\:+\:smooth}{Sum\:of\:True\:+\:Sum\:of\:Pred\:+\:smooth}=\:\frac{2.\:Intersection}{\sum\:Y+\:\sum\:\widehat{Y\:}}$$

### Area under the curve (AUC)

It measures the model’s ability to differentiate between disease classes. It is the area under the ROC curve.

14 $$\:\text{A}\text{U}\text{C}={\int\:}_{\text{R}\text{O}\text{C}}^{\:}\text{C}\text{u}\text{r}\text{v}\text{e}$$

ROC Curve: A graph showing the performance of a classification model at different.

threshold settings, ∫: Represents the integral (area) under the ROC curve.

### Precision and recall

The precision Evaluates the ratio of true positive predictions to the total predicted positive cases.

15 $$\:Pecision\:=\:\frac{True\:Positives}{Predicted\:Positives\:+\:\epsilon}$$

The recall assesses the ratio of true positive predictions to the total actual positive cases.

16 $$\:Recall\:=\:\frac{True\:Positives}{Possible\:Positives+\:\epsilon}$$

Predicted Positives = T P + F P, Possible Positives = T P + F N. True Positives (TP): The count of correct identifications of positive instances by the model, False Positives (FP): The model misidentifies negative instances as positive, False Negatives (FN): The model mistakenly categorizes positive instances as negative, ε: A small constant added to avoid division by zero.

### F1 score

The F1 score represents the harmonic average of precision and recall. For each single class in an image F1score is calculated independently.

17 $$\:F1=\:\frac{2.(Precision\:\times\:Recall)}{(Precision\:+\:Recall)}$$

## Results and discussion

**Fig. 10:**
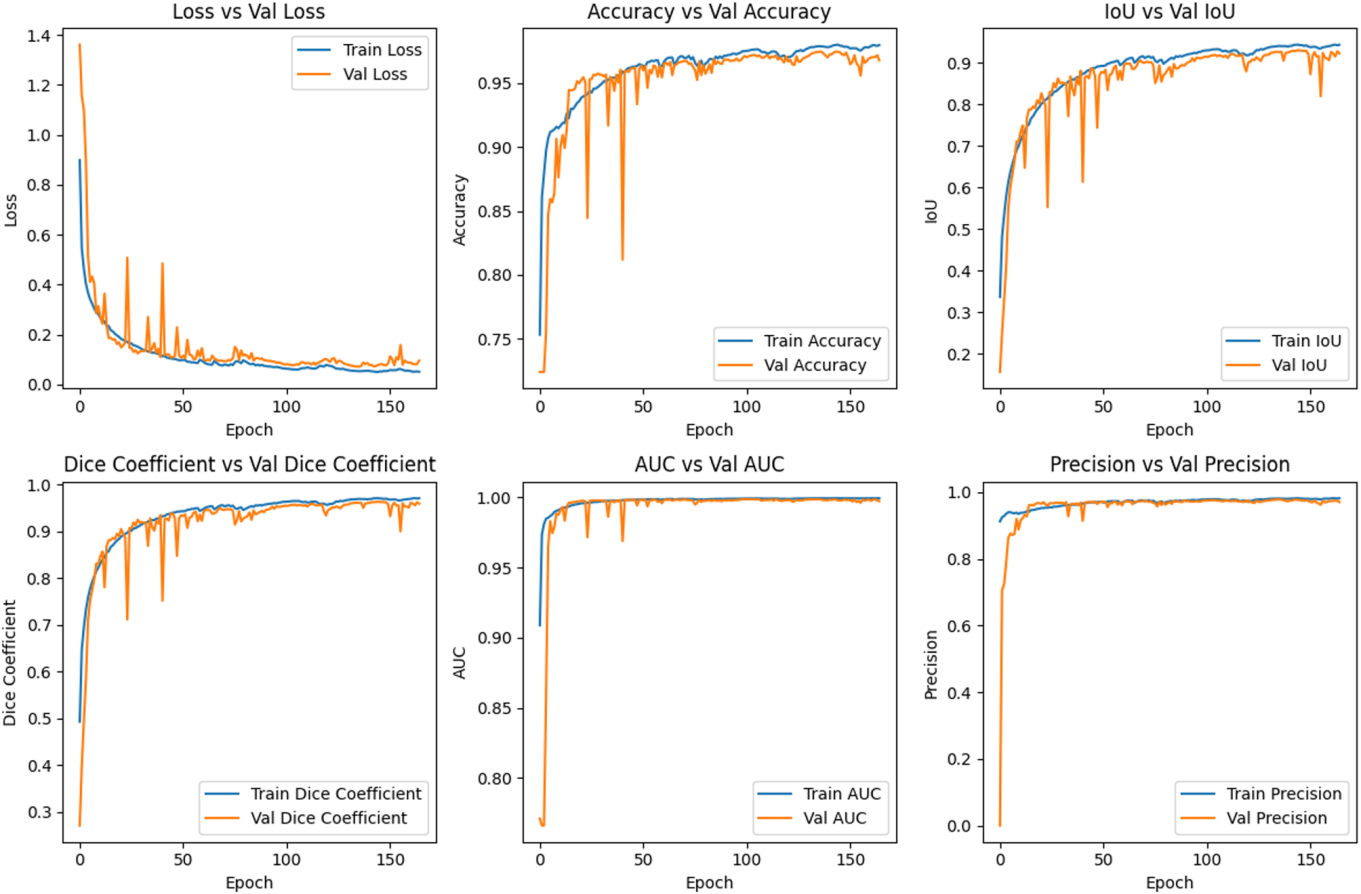
Shows Training and Validation graph of Accuracy, Loss, IoU, Dice-Coefficient, AUC, Precision.

Table 4 below demonstrates that a more efficient multiclass segmentation of diseased regions has been achieved. The segmentation results were evaluated using existing CNN models, considering metrics such as training and validation accuracy, loss, precision, recall, and IoU. The results clearly indicate the success of the proposed multiclass segmentation method, achieving a validation accuracy of 0.9755, a loss of 0.0660, a precision of 0.9779, a recall of 0.9738, and an IoU of 0.9308. Figure 10 presents graphs depicting the training and validation performance for various metrics, including accuracy, loss, IoU, Dice coefficient, AUC, and precision of the proposed model. Table 5 presents a detailed comparison of various U-Net-based architectures evaluated in this study. Each model’s performance is analyzed based on its Intersection over Union (IoU) accuracy, computational complexity (FLOPs), total number of parameters, and associated limitations. The computational complexity of the model is calculated using the parameter, floating-point operations (FLOPs). For any given convolution layer, number of FLOPs is calculated as two times the product of the kernel height, the output height, kernel width, output width and the total number of kernels present. For a fully connected convolution layer, no. of FLOPs is calculated as two times the product of output and input size. Layers such as BatchNormalization, activation functions like ReLU, LeakyReLU, and pooling contribute marginal FLOPs compared to convolutional operations. In this study, the FLOPs, parameter and IoU accuracy for each model variant were observed as follows: The standard Attention U-Net achieved an IoU of 0.9186 with a medium computational complexity of 21.1 GFLOPs and 26.48 million parameters. Incorporating transformer blocks slightly reduced the computational cost to medium-low of 14.6 GFLOPs and 25.7 million parameters, achieving an IoU of 0.9177. The introduction of residual connections and squeeze-and-excitation (SE) blocks led to a notable improvement in accuracy 0.9260 IoU, at the cost of increased complexity to medium – high of 34.7 GFLOPs and 37.9 million parameters. Finally, the proposed RSLLinked-TransNet model further optimized performance, achieving the highest IoU of 0.9308 while maintaining the same so it is medium-optimized complexity of 34.7 GFLOPs and parameter count to 37.9 million. Although it delivers superior segmentation tailored to specific leaf structures, its generalization ability to other domains may require further validation. These comparisons clearly demonstrate the trade-offs between model complexity, segmentation accuracy, and generalizability.

**Table 4:** Evaluation metrics obtained on proposed RSL Linked-TransNet and other UNet model. (Bold highlighted represent the best results)

Model	Accuracy		Loss		precision		Recall		IoU	
	Train	Val	Train	Val	Train	Val	Train	Val	Train	Val
U-Net (Attention)	0.9696	**0.9780**	0.0447	0.0823	0.9911	0.9688	0.9879	0.9638	0.9320	0.9186
U-Net with Transformer	0.9786	0.9654	0.0383	0.0827	0.9901	0.9683	0.9870	0.9636	0.9403	0.9177
Residual SE-UNet	0.9749	0.9642	0.0622	0.0798	0.9779	0.9775	0.9727	0.9698	0.9307	0.9260
**RSL Linked-TransNet** **(Proposed)**	**0.9917**	0.9755	**0.0244**	**0.0660**	**0.9927**	**0.9779**	**0.9927**	**0.9738**	**0.9580**	**0.9308**

**Table 5:** Comparison of models in terms of accuracy, complexity, parameter count, and limitations.

Model	Accuracy on IoU	Complexity / FLOPs	Total no. of parameter	Limitation
U-Net (Attention)	0.9186	Medium / 21.1 GFLOPs	26,481,169 with memory size of 101.02 MB	Struggles with diverse classes with fine details, lacks global context modeling.
U-Net with Transformer	0.9177	Medium - Low/ 14.6 GFLOPs	25,707,281 with memory size of 98.07 MB	Limited feature reconstruction for finer details.
Residual SE-UNet	0.9260	Medium- High / 34.7 GFLOPs	37,905,217 with memory size of 144.60 MB	Lacks effective handling of long-range dependencies, misses small disease details.
**RSLLinked-TransNet** **(Proposed)**	**0.9308**	Medium - Optimized / 34.7 GFLOPs	37,905,217 with memory size of 144.60 MB	Very few misclassification of closely resembling diseases.

**Fig. 11:**
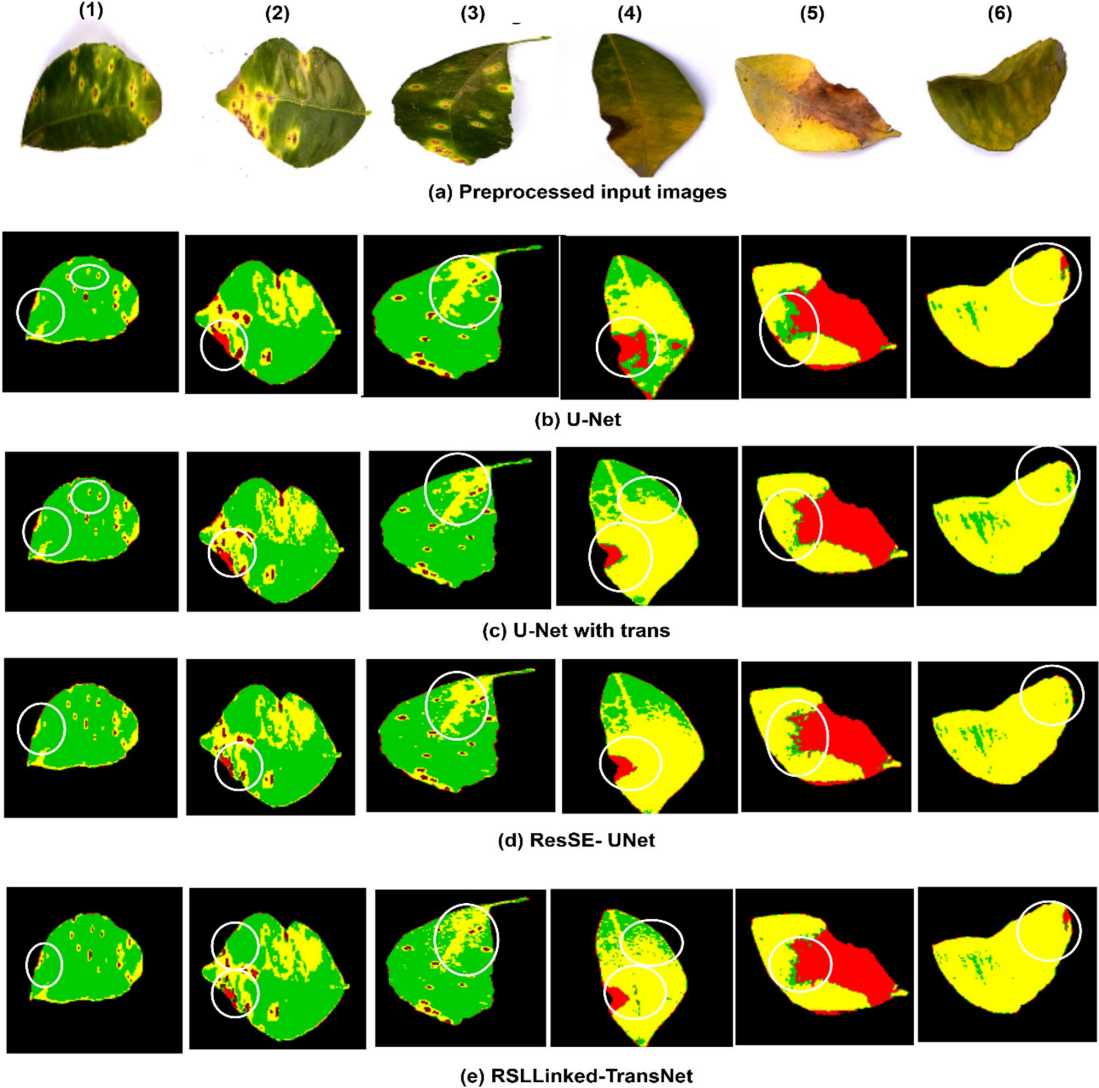
A visual comparison of various methods applied to the citrus validation dataset is presented. To highlight the differences between our method and others, the distinct areas are marked using white ellipses.

Figure 11 compares the results of the proposed RSL Linked-TransNet with those of UNet, UNet-Trans, and ResSE-UNet. The comparison highlights that the proposed model excels in capturing finer details, such as shades, textures, and regions of smaller disease classes. Additionally, it significantly enhances the processing of edges and boundaries for each class within the images. Further, the proposed model tackles some misclassifications like in Fig. 11, images b-1, c-1, d-1 regions are canker which are misclassified as blackspot; But in same figure image e-1, generated by the proposed model classifies the same image boundary regions as canker, thus correcting the previous misclassification. Another instance, in Fig. 11, image c-6, d-6, circled regions are misclassified as greening, which is again correctly classified as blackspot by the proposed model as seen in image e-6. In images b-2, c-2 and d-2 canker region is misclassified as blackspot, but the proposed model reduces this misclassification to the minimum as shown in the image e-2. Apart from this, images b-3, b-4, b-5 show the misclassified disease edges on the leaf, same was seen in c and d as well. The proposed model reduces this misclassification and detects fine-grained disease regions on the leaf, as seen in image e-3, e-4 and e-5, thus improving the classification of disease regions when compared with previous models.

**Fig. 12:**
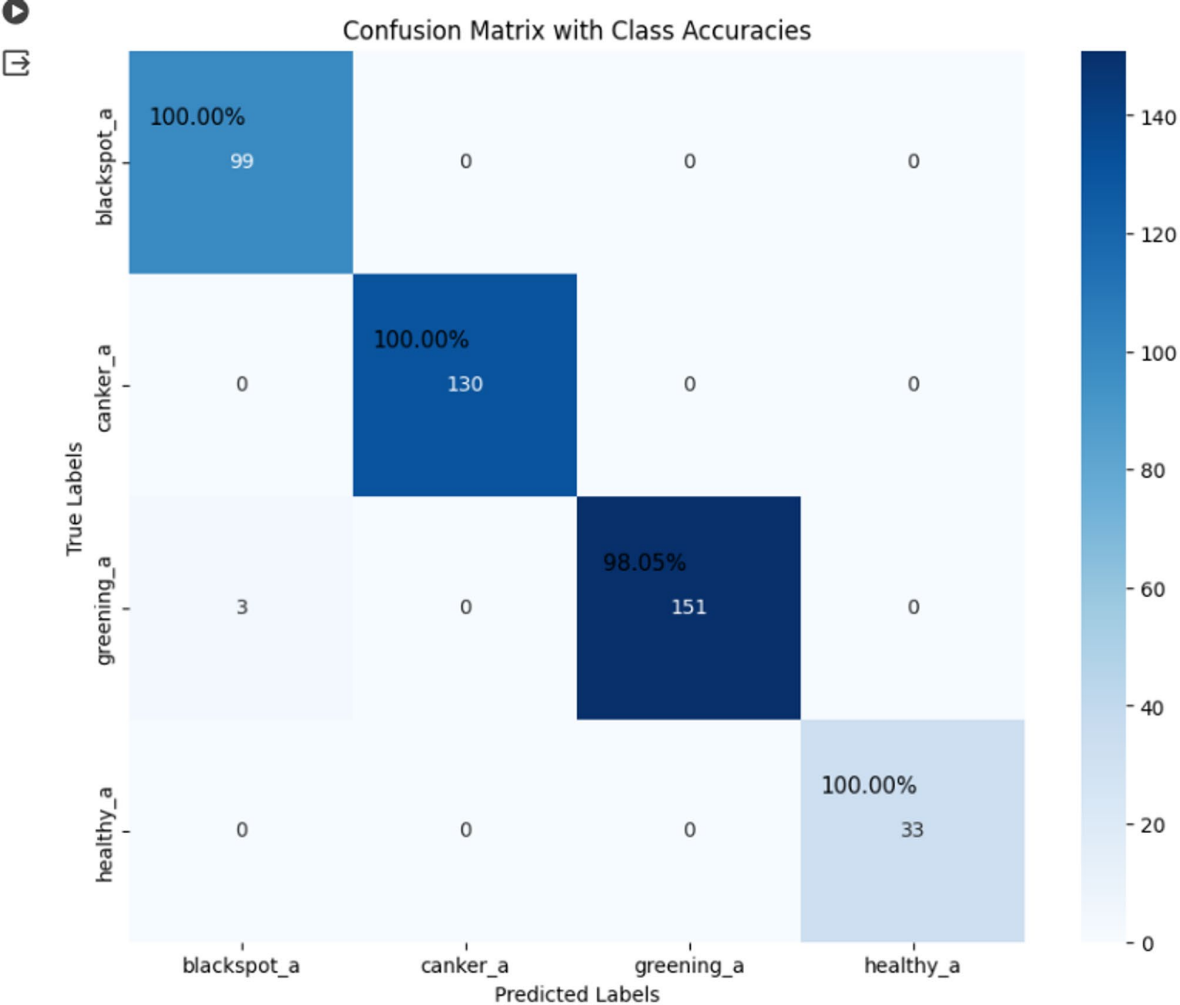
Confusion Matrix with each predicted class accuracy.

The proposed model achieves a mean F1 score of 0.7173 and a mean IoU of 0.75677 for each disease class in the segmented test images. Table 6 provides a detailed breakdown of the IoU and F1 scores for individual disease classes. Further the severity-based classification is done using a threshold. The value of the threshold is chosen for canker, blackspot, greening as 0.1, 1, 9 respectively. Figure 12 illustrates the confusion matrix, highlighting class accuracies for the identified prime disease class of the leaf. The matrix evaluates the model’s performance by comparing true class labels with the predicted labels for the prime disease. Correct classifications are represented in the diagonal cells, while errors are displayed in the off-diagonal cells. The model achieved 100% accuracy for Black Spot, Canker, and Healthy classes, and 98.05% accuracy for the Greening class, with minimal misclassifications. The colour intensity corresponds to the sample count, with darker shades indicating higher values.

**Fig. 13:**
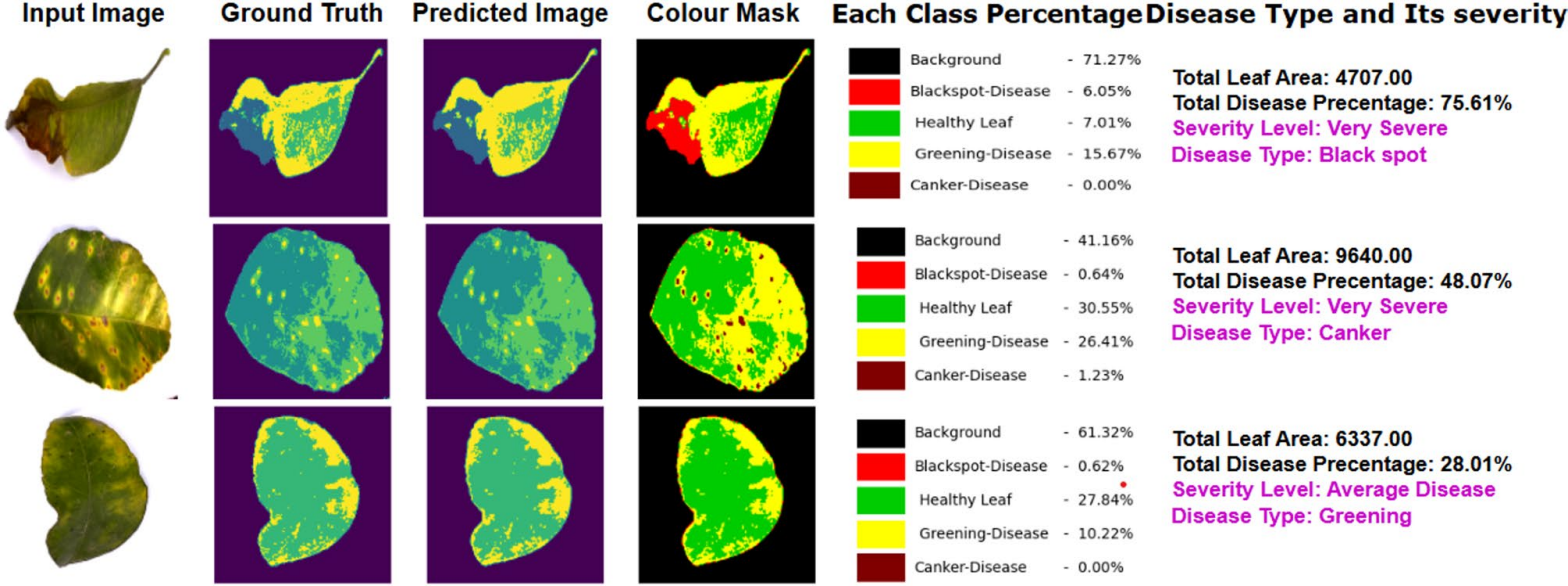
Sample output of the proposed model with each class percentage, severity type and classifying prime disease.

Finally, the severity level to indicate the quality of affected area is calculated. Here the affected area represents all the disease class regions in the image and the total leaf area are represent number of pixels in an image excluding background. From the infected area the severity level is calculated with the set of general thresholds. The value of the average, heavy, severe threshold is fixed. Figure 13 presents a detailed visualization of the segmentation results and their corresponding analyses. This comprehensive evaluation emphasizes the accuracy and effectiveness of the segmentation model in detecting, measuring various citrus diseases, assessing their severity levels and identifying the prime disease.

**Table 6:** IoU and F1 score obtained on each disease class in segmented test image.

Class	IoU	F1 score
Background	0.99472	0.99735
Blackspot	0.54779	0.67044
Leaf	0.67696	0.78995
Greening	0.86583	0.92499
Canker	0.69857	0.20414
**Mean**	**0.75677**	**0.71737**

## Conclusion

The RSL Linked-TransNet model is a novel approach proposed for multiclass segmentation, designed to accurately identify multiple disease classes within an image. The segmentation outcomes, along with the performance of individual disease classes, exhibit significant enhancements compared to existing models such as U-Net, U-Net Trans, and Residual SE-U-Net. These improvements are evaluated using metrics like accuracy, loss, precision, recall, and IoU. Additionally, the model determines the severity levels of infected areas by employing a dedicated algorithm for prime disease classification and severity assessment. From the results it is observed that the proposed method outperforms the other existing models and it can be effectively used for plant disease detection and severity based classification. In the future, the proposed method can be integrated with IoT devices to gather climate data, moisture levels, and other environmental parameters, enabling the development of a smarter and more sustainable solution. Additionally, it can be expanded to cover a wider variety of leaves and diseases using a larger dataset, with the inclusion of more disease classes.

## Data Availability

The authors are willing to share the experiment data to the interested researchers on request to the corresponding author.
